# Reaching the End-Game for GWAS: Machine Learning Approaches for the Prioritization of Complex Disease Loci

**DOI:** 10.3389/fgene.2020.00350

**Published:** 2020-04-15

**Authors:** Hannah L. Nicholls, Christopher R. John, David S. Watson, Patricia B. Munroe, Michael R. Barnes, Claudia P. Cabrera

**Affiliations:** ^1^Clinical Pharmacology, William Harvey Research Institute, Barts and The London School of Medicine and Dentistry, Queen Mary University of London, London, United Kingdom; ^2^Centre for Translational Bioinformatics, William Harvey Research Institute, Barts and the London School of Medicine and Dentistry, Queen Mary University of London, London, United Kingdom; ^3^Centre for Experimental Medicine and Rheumatology, William Harvey Research Institute, Barts and the London School of Medicine and Dentistry, Queen Mary University of London, London, United Kingdom; ^4^Oxford Internet Institute, University of Oxford, Oxford, United Kingdom; ^5^NIHR Barts Biomedical Research Centre, Barts and The London School of Medicine and Dentistry, Queen Mary University of London, London, United Kingdom; ^6^The Alan Turing Institute, British Library, London, United Kingdom

**Keywords:** machine learning, artificial intelligence, genome-wide association study, genomics, candidate gene, clinical translation, deep learning, data science

## Abstract

Genome-wide association studies (GWAS) have revealed thousands of genetic loci that underpin the complex biology of many human traits. However, the strength of GWAS – the ability to detect genetic association by linkage disequilibrium (LD) – is also its limitation. Whilst the ever-increasing study size and improved design have augmented the power of GWAS to detect effects, differentiation of causal variants or genes from other highly correlated genes associated by LD remains the real challenge. This has severely hindered the biological insights and clinical translation of GWAS findings. Although thousands of disease susceptibility loci have been reported, causal genes at these loci remain elusive. Machine learning (ML) techniques offer an opportunity to dissect the heterogeneity of variant and gene signals in the post-GWAS analysis phase. ML models for GWAS prioritization vary greatly in their complexity, ranging from relatively simple logistic regression approaches to more complex ensemble models such as random forests and gradient boosting, as well as deep learning models, i.e., neural networks. Paired with functional validation, these methods show important promise for clinical translation, providing a strong evidence-based approach to direct post-GWAS research. However, as ML approaches continue to evolve to meet the challenge of causal gene identification, a critical assessment of the underlying methodologies and their applicability to the GWAS prioritization problem is needed. This review investigates the landscape of ML applications in three parts: selected models, input features, and output model performance, with a focus on prioritizations of complex disease associated loci. Overall, we explore the contributions ML has made towards reaching the GWAS end-game with consequent wide-ranging translational impact.

## Introduction

A genome-wide association study (GWAS) examines a genome-wide set of genetic variants in a group of individuals to identify variants associated with a trait or phenotype. The goal of GWAS is to identify variants which show a statistically significant association with a phenotype. This enables guided functional investigation of the most likely causal variants and genes driving the genetic association, thus pinpointing genes and pathways of interest for disease diagnosis, drug discovery, and precision medicine.

As GWAS studies have scaled up to discover ever more disease variants (Evangelou et al., 2018; Giri et al., 2019; Nalls et al., 2019) it has become impractical to perform functional investigation on all disease relevant loci. This limitation arises in part due to variability in reporting of GWAS results, some studies report loci which have been independently replicated in a different cohort (the gold standard approach), and others do not. This reporting can question the confidence of some discovered loci, calling for a balance between stringent *p*-values to correct for multiple testing and false discovery, and conservative correction leading to false negative association. A compounding factor is also the need to differentiate causal variants or genes from other genes associated by linkage disequilibrium (LD), thus confounding the detection of causal genes within a locus – making it unclear which variants and genes warrant further analysis and potential functional study. This range of issues undermines the robustness of GWAS, and challenges the validity of downstream analyses and biological hypothesis development, critically undermining some of the major motivators for performing GWAS in the first place, such as target validation (Hurle et al., 2016). Ultimately this highlights the need for computational solutions to improve the signal to noise ratio of GWAS results and to highlight genes and variants that are most likely to be causal.

**FIGURE 1:**
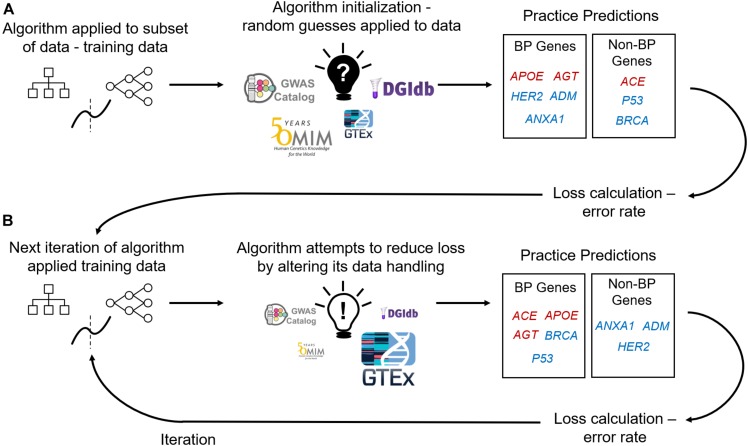
Supervised Machine Learning Algorithm Training. **(A)** Data containing labeled genes (e.g., genes labeled as causal or non-causal for blood pressure – BP) and columns of features describing those genes are input into a machine learning algorithm. Machine learning algorithms firstly initialize themselves by applying their rules to a subset of the data (deemed training data) and its features at random. E.g., an algorithm’s first practice iteration can involve assigning feature importance at random (importance denoted by size of feature image). The algorithm uses its feature initialization to classify genes into either affecting BP (red genes) or not affecting BP (blue genes). Algorithms then use the practice predictions to calculate loss (an error rate) and iterate over the data again with applying the previous iteration’s loss calculation to adjust feature handling **(B)**. With using the loss calculations the algorithm aims to improve predictive performance with each training iteration.

Machine learning (ML) has been one emerging branch of computational applications (alongside network analysis and tools such as text-mining) built to enhance GWAS performance and downstream interpretation (Seyyedrazzagi and Navimipour, 2017; Raj and Sreeja, 2018). Machine learning algorithms build mathematical models that are learnt from training data in order to make predictions or decisions. Machine learning consists of supervised, unsupervised, and reinforcement learning methods, with supervised and unsupervised learning being the most commonly implemented with GWAS data. Supervised learning provides ML algorithms with labeled training data and aims to infer a mapping function from the input variables to the output variable – or label for classification tasks (Figure 1). This mapping function may then be used to predict the labels of new “testing” data. Unsupervised learning, by contrast, has no response variable. Instead, the algorithm must attempt to find patterns in the data, such as clusters or outliers. When tailored for understanding GWAS data, ML predictions can provide an improved statistical foundation of evidence to support or improve GWAS results. For instance, ML in GWAS has been applied to identify loci, increase the statistical power of GWAS (Mieth et al., 2016), detect epistatic interactions (Leem et al., 2014), improve polygenic risk scoring produced from GWAS (Pare et al., 2017), and prioritize genes and variants on post-GWAS analysis (Vitsios and Petrovski, 2019). Here we will focus on the ML applications developed for post-GWAS prioritization.

The growth of GWAS over the past decade has identified thousands of associated loci, in September 2019 the NHGRI-EBI GWAS catalog contained 161,525 variant-trait associations from 4,298 publications^1^. Thousands of variant associations can now be found within a single complex disease, such is the case for inflammatory bowel diseases (IBD) with 1,829 variant associations and schizophrenia with 3,069 variant associations (see text footnote 1). In the case of blood pressure (BP) with 5,148 associations (see text footnote 1) 2,293 genes are implicated (Evangelou et al., 2018; Giri et al., 2019), these represent almost 10% of the known gene complement and 5.82% of the genome by LD alone. These results represent an important insight into the complex systems regulating BP and offer a basis for a better understanding of BP biology and the personalization of hypertension treatment. However, this knowledge still has great potential to confound understanding. Based on the simplifying assumption that each locus is driven by only one gene (whereas gene cluster associations are also possible), if we subtract 901 loci reported by Evangelou et al. (2018) from 2,605 genes mapping to these loci, 65.4% of “associated” genes can be expected to be unrelated to BP. This level of signal to noise, still presents a considerable problem to the formulation of an efficient follow up strategy.

Individual GWAS loci have already shown the potential for large scale prioritization by providing novel biological insights and potential drug targets and drug repositioning opportunities (Sanseau et al., 2012). For example, a GWAS on BP found associations in the *SLC5A1* gene. The association of *SLC5A1* with BP and its role as a target of a type 2 diabetes drug, canagliflozin, highlights the opportunity to re-purpose drugs for treating hypertension (Evangelou et al., 2018). Currently, research has shown only 38% of essential hypertension patients have effective treatment (Banegas et al., 2011). Similarly, IBD and schizophrenia both currently have lacking treatment options alongside their thousands of associations (Danese, 2012; Leucht et al., 2013) – suggesting that a path to improved therapeutics for complex diseases may lie within the associated loci and the biological functions contained within them.

Defining functional impact of associated variants is a unique challenge in itself, but it is subsumed by a greater problem. Although it is possible to predict functional impact with some confidence in coding regions and to a lesser extent in non-coding regions, differentiating variants and inferring causality is very challenging without further laboratory investigation. For example, BP associations found in several *SMAD* family genes and the *TGFβ* gene, which collectively participate in the TGFβ pathway, led to the suggestion that these may affect sodium transport in the kidney and ventricular remodeling (Evangelou et al., 2018). However, multiple genes impacting the same pathway raise the question of which gene should be functionally investigated first. Usually the evidence is not strong enough to warrant laboratory investigation of all the associated genes in a particular pathway. The follow-up GWAS laboratory studies to date have developed without a standardized method for selecting causal genes and consequently they are likely to be susceptible to personal or “cherry picking” bias. These issues highlight the need for a pipeline that methodically triages variants and genes based on their likelihood of affecting a trait. Only then, will there be consistency in follow-up of genetic results using functional analysis with minimized risk of investigating false positives or low impact genes. The standardized *in silico* identification of the most likely causal genes at a genome scale may be an opportunity to gain higher level systems insights into trait biology. This in turn may help to fine-tune ML algorithms, as seen with research using ML variant prioritization as a feature fed into gene prioritization (Khan et al., 2018).

**FIGURE 2:**
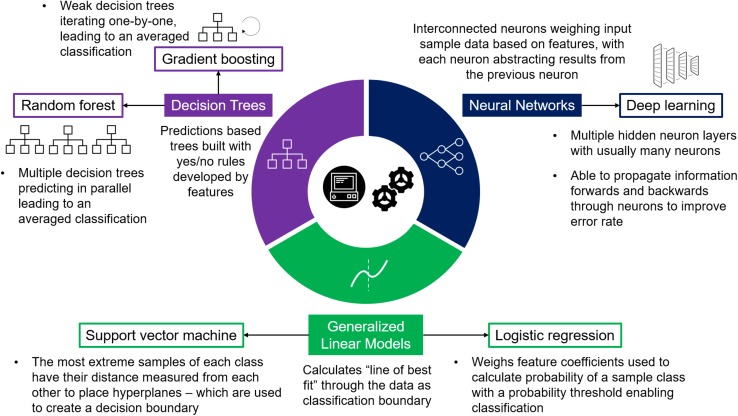
Supervised Machine Learning Models. Diagram detailing three machine learning model bases used in supervised learning, each providing varying algorithms most commonly used in post-GWAS prioritization.

The development of systematic prioritization post-GWAS using ML has been researched as early as 2007 (Lewinger et al., 2007). Since then several computational methods for prioritizing GWAS associated loci have been developed with growing attention on ML applications (Fridley et al., 2011; Gagliano et al., 2015; Raj and Sreeja, 2018; Wu et al., 2018). ML for prioritizing GWAS results has used common models (Figure 2) such as logistic regression, decision tree classifiers such as – e.g., gradient boosting machines (GBM) and random forests (Wang et al., 2013; Oh et al., 2017), – and support vector machines (SVM; Vitsios and Petrovski, 2019), with more recent advances including deep learning models (Khan et al., 2018; Zhou et al., 2018).

An increasing number of studies are investigating how ML can be tailored to locus prioritization across diseases, but the ML pipelines for GWAS prioritization are mainly limited by the range and quality of training data. In order for ML models to present reliable guide-posts for post-GWAS research, a critical assessment of developing methods is needed – as the most recent systematic and literature reviews of post-GWAS prioritization cover few ML studies in comparison to other prioritization methods (Seyyedrazzagi and Navimipour, 2017; Raj and Sreeja, 2018). Here we will review the current landscape of ML applications for post-GWAS prioritization, and how ML can aid reaching the end-game for GWAS, which we define as a state where all common population variation with impact on a trait is identified; providing solid biological insights and mechanisms with reliable translational capability.

## Machine Learning Models

GWAS prioritization as a classification problem has been approached using both simplistic and complex models (Table 1) depending on the problem requirements and data available. Primarily five types of models have been implemented: logistic regression, SVM, random forest, gradient boosting, and deep neural networks (Figure 2), each with varying advantages and disadvantages (Table 2). Logistic regression is a commonly applied statistical method that when used with categorical variables can be contemplated as a generalized linear model. In a logistic regression, it is typical to apply a regularization term – e.g., L1 (the sum of the absolute value of feature weights) and L2 (the sum of squared feature weights) – that introduce some bias while reducing variance, thereby improving predictive ability (Demir-Kavuk et al., 2011). Isakov et al. (2017) used elastic net logistic regression (Zou and Hastie, 2005) which combines L1 and L2 penalties to prioritize IBD genes. This method performs both variable selection (L1), and shrinks coefficient sizes to reduce variance (L2) (Ogutu et al., 2012). Regularized logistic regression with elastic net aims to minimize the “curse of dimensionality” – where data has a larger number of features than samples – which is a particular blight on GWAS. For example, Isakov et al. (2017) used data consisting of 314 positive genes and 1,736 negative genes each annotated with 1,027 features. By applying logistic regression with elastic net they could then select the best data for their models (309 features selected which are predominantly from biological ontologies). However, due to the growing size in genetic data, and the broader range of features becoming available to describe genes and variants, the increased computational demand requires more advanced models.

**TABLE 1:** Curation of machine learning studies applied to post-GWAS prioritization of variants and genes.

**PMID**	**Models description**	**Methods description**	**Data and assessment descriptions**
30692607*	LR; Genes – Crohn’s disease	Uses backward stepwise regression to build significant expression datasets (with emphasis on epigenetic data) to give prediction in combination with genotype data. Expression data reduces the uncertainty of smaller effect loci shown in fine-mapping and prioritization was followed-up with protein network analyses for validation	10-fold cross validation (2,000 genes per fold)
25935003*	LR; Genes – Crohn’s disease	Combines GWAS results with gene expression features and whether genes are associated with other autoimmune diseases to better identify disease-related genes. More powerful for prioritizing rare missense variants	Cross-validation performed. 50:50 training:testing ratio. Training iterated 500 times. 54 Crohn’s disease genes used as positively labeled training genes
29407288	SVM, LASSO, classification-regression trees; Variants – major depressive disorder and adverse drug response (duloxetine)	Models used features selected by LASSO regression and classified variants based on a clinical depression scoring defining drug response and remission	Dataset size: 186 patients. Nested 5-fold cross-validation. 80:20 training:testing ratio
21317188*	SVM, RF; Variants – arthritis and T1D	Compares support vector machine and random forest performance to chi-squared ranking	Dataset size: 452,176 T1D SNPs 63 arthritis SNPs
31779641	RF; Variants – intronic variants associated to cellular sensitivity to clofarabine-induced cytotoxicity	Focuses on integrating splicing data features with other types. Validates model prioritization with laboratory follow-up – limited by technical noise during laboratory work	3-fold cross-validation. Training data size: 6,676 variants. Testing data size: 1,222 variants
24564704*	Parallel RF Regression; Variants – brain structure and function. Alzheimer’s disease GWAS	Designed to run on large Hadoop clusters, including those available through cloud computing. Multivariate applications not available on Hadoop	Each tree bootstraps to form training data (63.2%) with out-of-bag samples for test data. 500 simulated datasets
28592878*	RF Hyper-ensemble; Non-coding variants – curated mendelian diseases	Addresses class imbalance via resampling using simultaneous oversampling of minority class and undersampling of majority class. HyperSMURF can detect disease variants nearby to non-disease variants	10-fold cross-validation partitioning variants into chromosomal bands so no variants had same location, gene or disease in training and testing. GWAS total size approximately 2,000 variants
25633252*	GB; Genes – cardiovascular diseases and traits	Explored prioritization of 38 phenotypes (predominantly cardiovascular). Each tree within model updates a log-odds of disease association per gene. GWA-prediction assigns scores to genes in loci based on reasonings (transcription sites, experimental evidence, etc.) to identify likely positives which are used in training for phenotypes with GWAS training data	Six rounds of 8-fold cross-validation. Seventy percent of loci as positive training examples with matching numbers of negative samples
30591030*	LR and DL; Genes and variants – schizophrenia and autism	Performed variant prioritization which fed into gene prioritization. Variant prioritization used eQTL and pathogenic scoring data features. Gene prioritization used the variant rank in combination with genotypic data. Used to prioritize an individual’s variants and genes and can be re-applied to GWAS data	10-fold cross-validation on four training and test sets
28795970	LR with elastic net, RF, SVM with polynomial kernel, extreme GB; Genes – inflammatory bowel diseases	All genes in dataset were annotated with 1,027 features. 16,390 genes scored and classified, with prediction as a score between 0 and 1. Models evaluated separately and together in combined performance score	5-fold cross-validation repeated 10 times. Training data: 314 positive genes and 1,736 negative genes
30013180*	DL – ExPecto; Variants – publicly available GWAS for four immune diseases	Data profiling >140 million promoter-proximal mutations allowed for deep learning to predict variant effect, with effect feeding into the prioritization of SNPs	Dataset size: 390,085 variants. Whole-chromosome holdout of chromosome 8 with 990 genes – using these genes for testing
30859622	LR with stochastic gradient descent, SVM, RF, K-Nearest Neighbors; Genes – colorectal cancer	Used a network approach – collecting both global and local data to create an epistasis network. Topology of the network was then used as features in machine learning, with different types of feature selection compared, to prioritize genes biologically relevant to colorectal cancer	Dataset size: 185,180 SNPs. Training on 90% of the dataset with 10-fold cross validation
doi: 10.1101/655449*	SVM, RF, extra trees, GB, extreme GB, DNN and a stacking classifier with four base classifiers (RF, extra trees, GB and SVM) followed by a DNN in the second layer. Genes – chronic kidney disease, amyotrophic lateral sclerosis, epilepsy	Models applied with positive-unlabeled learning – stochastic semi-supervised learning. Explored combinational impact of all models, and chose best performing model for each disease. There was a dependency on existing patterns – beneficial for finding new causal associated genes which may impact known mechanisms	10-fold cross validation. Gene samples: 25,000 for chronic kidney disease, 17,000 for epilepsy and 79,500 for amyotrophic lateral sclerosis
21687685	Bayesian latent variable model; Variants – ovarian GWAS	Used features about a SNP to estimate a latent quality score, with SNPs prioritized based on the posterior probability distribution of the rankings of latent quality scores. Incorporated the uncertainty of the ranking into the prioritization via probability calculation	NA
23369106*	Genetic algorithm; Variants – select OMIM diseases	Algorithm estimates feature weights to characterize SNPs related to an input dataset of genes, biological processes or GWAS results. Users can select features and assign a custom relevance and model relies on data mining of public data	Leave one out cross validation – single disease in the set used to validate (repeats for each disease)
29874547*	Network representation learning (random walk); Genes – Parkinson’s, RA, Crohn’s, Ulcerative Colitis, CAD, T2D	Unsupervised model learns embeddings of genes from multiple gene networks and develops hierarchical statistical model to integrate the learned embeddings of genes with GWAS summary data. Gene-level *p*-values infer each gene’s posterior probability of association, which is in turn used for gene prioritization. Lack of direct biological interpretations available for the learned embeddings of genes	NA
21977986*	Multi-task learning ProDiGe; Genes – 265 diseases and 936 associations	Model learns from positive and unlabeled examples. The model shared information across diseases to improve the predictive performance for diseases with minimal positive labeled genes. The information shared is weighed depending on similarity of one disease to another	Training set: at least one known disease gene in training data. Training data per disease >11 genes. Leave one out validation on select diseases
26504140*	Unsupervised model – bayes classifier – GenoWAP; Variants – schizophrenia and Crohn’s disease	Unsupervised learning – integrates GenoCanyon (their previous model) functional prediction and GWAS *p*-values. Reduce noises caused by linkage disequilibrium and rescues marginal signals in GWASs with insufficient sample sizes	NA
27058395*	Unsupervised model – bayes classifier – Genoskyline; Variants – schizophrenia and coronary artery disease	Successor of GenoWAP model, building from it by using annotations integrating tissue-specificity. Customizable with researchers able to input many feature annotations. Whilst tissue-specific it also lacked data from all tissue types	NA

*Software/code available; LR, logistic regression; RF, random forest; GBM, gradient boosting machine; SVM, support vector machine; DL, deep learning; DNN, deep neural networks; ET, extra trees; GWAS; genome wide association study; SNP; single nucleotide polymorphism; CAD, coronary artery disease; T1D, type 1 diabetes; T2D, type 2 diabetes.

**TABLE 2:** Comparison of machine learning model performance. Comparison of the most common models used in post-GWAS prioritization including performance metrics, comparing metrics of each model’s highest performance score per study.

**Models**	**PMID**	**Best performance**	**Model advantages and disadvantages**
Logistic regression	25935003	0.94 (AUC) – Crohn’s disease	Advantages: - Easy to implement - Efficient to train - High interpretability - Can act as a benchmark for exploring more complex algorithms Disadvantages: - Difficulty recognizing complicated data patterns - Difficulty handling large datasets
	28795970	0.775 (ROC) – inflammatory bowel diseases	
Random forest	28592878	0.635 (AUCROC) – curated Mendelian diseases	Advantages: - It can handle large data with higher dimensions - Ensemble method reduces overfitting by several models testing multiple hypotheses Disadvantages: - Many parameters to tune, affecting computational efficiency - Ensemble method lows interpretability
	31779641	0.96 (AUCROC) – cellular sensitivity to clofarabine-induced cytotoxicity	
	21317188	0.81 (AUC) – T1D	
	28795970	0.80 (ROC) – inflammatory bowel diseases	
	doi: 10.1101/655449	0.85 (AUC) – average between all diseases	
Gradient boosting	28795970	0.783 (ROC) – inflammatory bowel diseases	Advantages: - High power performance - Flexible with several parameter tuning options - Ensemble method reduces overfitting by several models testing multiple hypotheses Disadvantages: - Reliance on high quality training data - Many parameters to tune, affecting computational efficiency
	doi: 10.1101/655449	0.848 (AUC) – average between all diseases	
	25633252	0.959 (ROC) – HCM	
Support vector machine	28795970	0.786 (ROC) – inflammatory bowel diseases	Advantages: - Computationally efficient - It handle can handle large data and high dimensions Disadvantages: - Does not provide class probabilities - Difficulty to interpret
	29407288	0.66 (Accuracy) – major depressive disorder and adverse drug response (duloxetine)	
	doi: 10.1101/655449	0.832 (AUC) – average between all diseases	
Deep neural network	30013180	0.815 (AUCROC) – lymphoblastoid expression	Advantages: - Recognizes patterns in large complex data - High power performance - Able to handle noisy data Disadvantages: - Difficulty to interpret - Computationally expensive requiring GPUs for high power performance

*AUC, area under curve; GPU, graphics processing unit; ROC, receiver operating characteristic; T1D, type 1 diabetes; HCM, hypertrophic cardiomyopathy.*

Seven out of 19 ML models for post-GWAS prioritization curated in this review (Table 1) are ensemble models, namely random forests and gradient boosting. Ensemble methods combine multiple models to improve performance and are ideal for heterogenous GWAS data. Deo et al. (2014) developed a GBM (OPEN – Objective Prioritization for Enhanced Novelty) for prioritizing causal genes in multiple diseases. They used data comprising of more than 40,000 genomic features from public databases [Gene ontology (GO), Mouse Phenotype database, Human Phenotype Ontology (HPO), and Online Mendelian Inheritance in Man (OMIM)] aiming to benefit from unbiased features. GBM is a tree-based model, with tree branches performing yes/no decisions leading to a sample’s classification (Natekin and Knoll, 2013). GBM operates one tree at a time, attempting to optimize with each tree. Deo et al. (2014) made accurate predictions with GBM identifying genes affecting cardiovascular disease (CVD) related traits. Performance was measured by the area under the receiver operating characteristic curve (AUROC), with values ranging between 0.75 and 0.9 across traits (Deo et al., 2014). The model’s consistently high scores are due to the ensemble methods providing the opportunity for predictive mistakes to be removed in aggregate, due to multiple models testing different hypotheses and taking an average, expanding the representational space of a classification problem (Dietterich, 2000). This is seen with gradient boosting across research, with the model known for reducing bias and variance and offering improved accuracy (Natekin and Knoll, 2013). However, there is also a need to benchmark model performance, as whilst ensemble models are reliable, a singular approach into a novel classification problem provides a risk of unnoticed overfitting – which is also a known issue for gradient boosting depending on regularization techniques used.

Vitsios and Petrovski (2019) built a semi-supervised learning framework in which they benchmarked seven models (random forest, extremely randomized trees, GBM, extreme gradient boosting, SVM, deep neural networks, and a stacking classifier using all models) to prioritize genes for three diseases – amyotrophic lateral sclerosis, chronic kidney disease and epilepsy. In total they used data containing more than 1,200 features describing tens of thousands of genes for each disease. They found that random forest was the top-performing classifier, with this ensemble model consisting of multiple decision trees predicting in parallel (Breiman, 2001). Gradient boosting was the second most accurate, showing the high performance of tree-based ensemble classification. However, the AUCs between all algorithms were deemed too similar to conclude one model out-performed all others across datasets. These results were also supported by comparison with a combined framework using all models in prioritization, the stacking classifier, ensuring the highest reliability in the chosen classifier for each disease (Vitsios and Petrovski, 2019). Kafaie et al. (2019) aimed to prioritize genes associated with colorectal cancer comparing various models (SVM, random forest, logistic regression with stochastic gradient descent, and K−nearest neighbors). They found that logistic regression was the highest performing ML model – emphasizing that a classification problem may require simpler solutions.

Besides ensemble learning and logistic regression, SVM is also consistently used within studies performing benchmark comparisons (Roshan et al., 2011; Isakov et al., 2017; Maciukiewicz et al., 2018; Vitsios and Petrovski, 2019). SVM aims to plot a decision boundary between groups by measuring hyperplanes – based on the distances between the most extreme samples of each classification group (Smola and Scholkopf, 2004; Figure 2). However, within benchmarking studies, SVM has not shown itself to be a top-performing model. For example, Vitsios and Petrovski (2019) found it had the lowest AUC (0.83, only slightly lower than the top-performing random forest at 0.85) of their seven models, while Kafaie et al. (2019) found SVM performed better than random forest yet worse than logistic regression. The varying performance of SVM also highlights the importance of input data, as Kafaie et al. (2019) were one of the only studies to focus on comparing feature selection methods as well as models. Kafaie et al. (2019) found SVM performed well given certain features, whilst in comparison logistic regression had a more stable high performance regardless of external selection, emphasizing the value of logistic regression’s internal feature selection via regularization.

Deep learning has also been explored for prioritization, this method can increase sensitivity in larger datasets due to the methods ability to incrementally capture abstract representations of high-level information. In general, this is beneficial for GWAS prioritization where the data is growing dramatically in size and heterogeneity with increasing annotations post-GWAS, and also has few labeled samples (known disease causing variants/genes) for supervised learning. Deep learning becomes advantageous in this scenario as it identifies complex patterns via supervised and unsupervised learning from large datasets (Najafabadi et al., 2015) and can be applied for further insights into GWAS data. However, whilst deep learning enables the consideration of millions of parameters, its application to date has mostly flourished in image classification and natural language processing (Zeng et al., 2018;Aung et al., 2019; Hampe et al., 2019), requiring an investment in its development and benchmarking with traditional models for developing GWAS application. A deep neural network (ExPecto) applied by Zhou et al. (2018) prioritized causal variants for immune-related diseases using sequence-based features. This dataset contained more than 140 million promoter-proximal mutations, and allowed for the unidirectional flow of information from base-sequence to functional predictions which enabled variant prioritization. To approach this large dataset ExPecto applies spatial transformation to the data, weighting transformations based on transcription start site distances. This was performed on a tissue-specific basis of over 200 tissues (Zhou et al., 2018), providing hundreds of features for the model to process. ExPecto is also able to perform pattern recognition and prioritization of rare and unobserved variants. However, whilst models are selected based on their suitability to the data, performance can also be dependent on class balance and data quality available.

Predominantly, ML studies use cross-validation to ensure a reliable estimate of model performance. However, with GWAS data commonly lacking functionally validated disease causing variants and genes, there are minimal learning opportunities for supervised models. Oversampling or undersampling techniques can be used to address class imbalance. Schubach et al. (2017) developed a hyper-ensemble model (hyperSMURF) using random forests with imbalance-awareness by using both under- and oversampling. By balancing the training data classes, and exposing the base learners in the hyper-ensemble to different training datasets, the random forests are able to diversify their understanding of the data, improving accuracy regardless of data size. Using hyperSMURF they prioritized thousands of GWAS variants annotated with 1,842 features. Their sampling techniques created balanced training data, where the original GWAS data had a 1:700 label imbalance. However, oversampling techniques develop synthetic samples based on example data points to increase the minority class size, which can create overfitting. Schubach et al. (2017) addressed this by preventing example variants of the same location/gene to occur in the training and test sets, minimizing the oversampling bias.

Whilst only one post-GWAS prioritization study has focused on class imbalance (Schubach et al., 2017), several have targeted data quality with a focus on data labeling. For example, positive unlabeled learning is semi-supervised learning with only positive labeled examples, a common occurrence for GWAS data where only a few causal genes have been functionally validated. For positive unlabeled learning overfitting is avoided using approaches such as classing unlabeled samples as negative and bootstrapping random samples. Vitsios and Petrovski (2019) applied positive labels to disease genes from the HPO with further validating clinician confirmation, and treated any unlabeled genes as negative samples. They then conducted random sampling of positive and unlabeled samples, aiming to equalize the ratios of the positive and negative genes to expose their models to a balanced dataset. Mordelet and Vert (2011) also applied their model (ProDiGie) using positive unlabeled learning. Whilst they only had minimal positive samples per disease, the model shared information across diseases – enabling it to use information from causal genes for closely related diseases in prioritization. Despite these benefits, positive unlabeled learning is limited by prior knowledge of known causal genes, leading to potential false negatives, and unlikely scenarios for a model to prioritize genes in novel mechanisms.

Overall, there is a need for benchmarking in order to select the model best suited to the data, and for post-GWAS prioritization the optimal model currently varies across diseases without a one-size-fits-all winner. An optimal model also hinges on data size and quality for reliability and performance, with studies varying in data size and choice of features – from using hundreds of selected features (Isakov et al., 2017) to others exploring tens of thousands (Deo et al., 2014). Further *in silico* methods need to address these aspects of ML, the lack of functionally validated associated genes at the disposal of ML, and how features are used in order to build a model tailored to post-GWAS prioritization.

## Feature Curation

To fine-tune a model, researchers must perform data curation and feature quality control to achieve the best possible performance. GWAS associations are typically annotated to a wide range of biological annotations. Biological features range from eQTL (expression quantitative trait loci), RNA, epigenetic, and protein data to describe a variant or gene’s functionality. For example, several studies use eQTL data, providing tissue-specific and population-specific insight, with researchers noting the use of eQTLs can improve the ability for models to distinguish single causal genes within a locus (Deo et al., 2014). For example, Ning et al. (2015) built a logistic regression for prioritizing Crohn’s disease associated genes. They found that integration of eQTL data with GWAS data provided an overlap of information between the two that strengthened model performance. Furthermore, the cataloging of eQTLs mapped to non-coding RNA provides a better insight into how non-coding RNA affects gene expression (Branco et al., 2018), increasing the strength of regulatory information at the disposal of ML models. The growing integration of related biological features suggest this will provide clearer insight for models to be able to pinpoint the most likely disease causing genes in a locus (Branco et al., 2018; Dai et al., 2019).

Other features used by studies are those provided by GO and Kyoto Encyclopedia of Genes and Genomes (KEGG) pathway database. Merelli et al. (2013) built SNPranker using terms from GO and KEGG to prioritize variants across diseases via a genetic algorithm. This model focuses on user-guided optimization, which is beneficial as SNPranker also takes features from data mining, allowing the researcher to adjust feature weights to minimize bias. Merelli et al. (2013) also focus on sharing information across ontologies, illuminating similar genes to those with known functional causality, indicating that this can grow the causal gene list (Merelli et al., 2013). Despite this possibility of increasing the model’s training data, expanding a list of causal genes based on known biological processes alone is likely to create susceptibility to bias and weaker model performance – as the model is then less able to prioritize loci within novel systems which may be affecting a phenotype.

The use of other biological features, e.g., RNA and epigenomic features, has also grown in recent years. These features may provide further insights into associated loci located in non-coding regions. For example, researchers developed and combined models GenoWAP and GenoSkyline (Lu et al., 2016a; Casas et al., 2005). Both methods use unsupervised learning – GenoWAP performs GWAS prioritization and GenoSkyline integrates tissue-specific and epigenomic annotations for predicting tissue-specific functional regions. They found these annotations showed both functional and non-functional tissue-specific variants were enriched, suggesting LD between variants in both regions (Lu et al., 2016b). For example, one schizophrenia associated locus within an intergenic region, upstream of *MMP16*, had high prioritization by GenoWAP in brain tissue (Lu et al., 2016b). This result is then augmented as GenoSkyline predicted that this locus plays a role in the functional regions downstream, offering new targets for further research. However, they concluded that their results can be improved with cell-specific data. Since GenoWAP, the ExPecto tool was built to make cell-type-specific predictions with high accuracy which it uses for variant prioritization, providing a novel method for generating cell-specific data *in silico* (Zhou et al., 2018). Whilst this method of predicting cell-specific data is disadvantageous to manual curation, the systematic collection of cell-specific data is in development and standardized resources have not been widely applied to post-GWAS analysis. Methods such as ExPecto provide a starting point for cell-specific curation and also a potential benchmark for the manual curation as it develops.

Alongside general biological characterization, disease-specific data is gradually increasing, further enabling accurate prioritization of GWAS associated loci. Vitsios and Petrovski (2019) for example prioritized chronic kidney disease genes, using annotations from the Chronic Kidney Disease database among their features to improve stratification. Algorithmic scorings are also used for prioritization (e.g., Eigen, CADD, DANN, GWAVA, DeepSea). These scorings predict pathogenicity of variants based on their expected functional consequences, and have been used to aid variant prioritization, however, to the best of our knowledge this is only been demonstrated by Khan et al. (2018), requiring further exploration into their benefits as features in ML prioritization.

Beyond data collection, studies also need to consider feature importance and feature selection to gain an understanding of models “under-the-hood” This is often a part of why researchers choose L1 regularized logistic regression, which automatically performs feature selection. Several studies have used logistic regression, such as Isakov et al. (2017) with using the elastic net, who found positive feature coefficients (predicting causal genes) were highest for immune and inflammatory response features from GO. Recently Gettler et al. (2019) also used logistic regression – as part of their gene prioritization regression model (GPRM) – to prioritize genes for Crohn’s disease. While Gettler et al. (2019) do not discuss the impact of feature importance, they note that GO enrichment analysis showed immune and inflammatory genes were significantly enriched. This enrichment is to be expected from an autoimmune disease, however, it also suggests validation for the feature importance found by Isakov et al. (2017). Maciukiewicz et al. (2018) applied L1 logistic regression to identify significant features, and followed-up with SVM for predicting causal variants for duloxetine response in major depressive disorder. They found a non-coding RNA annotation had the largest positive coefficient. However, unlike the study of IBDs, Maciukiewicz et al. (2018) is the first prioritization study to focus on their drug response phenotype, requiring further work to validate feature importance and begin to suggest how that may fit into biological understanding of GWAS results. There is also work focused primarily on improving feature selection for GWAS data (Szymczak et al., 2016; Nembrini et al., 2018). For example, random forests provide feature importance measures and have been investigated by Szymczak et al. (2016). They developed a recurrent relative variable importance measure from random forest to rank important variants in GWAS. This focus on feature importance developed a useful tool for highlighting loci deserving of functional-follow up and could be used to reduce false positive GWAS results (Szymczak et al., 2016). The only other study investigating feature importance in prioritization has been SNPranker, with Merelli et al. (2013) finding epigenetic features (namely enhancer, CpG islands, and DNase cluster data) had the highest importance for default prioritization. Additionally to a model’s internal feature weightings, permutation is also able to provide feature importance, doing so for any model by shuffling feature values and viewing model error rate. Vitsios and Petrovski (2019) use permutation via the boruta algorithm, which creates synthetic features from random permutation to weigh the importance of original features and remove any unimportant annotations. For all studies incorporating feature selection or importance they note an improvement in model performance or understanding of their predictive reasoning.

## Prioritization of Variants and Candidate Genes

Prioritization methods post-GWAS have had development for several models that aim to be applicable for multiple diseases – e.g., ExPecto (Zhou et al., 2018), GenoWAP (Lu et al., 2016b), HyperSMURF (Schubach et al., 2017), and SNPRanker (Merelli et al., 2013). For example, ExPecto used all publicly available GWAS data for prioritizing variants for Crohn’s disease, ulcerative colitis, Behçet’s disease, and hepatitis B virus (Zhou et al., 2018). On prioritization they found highly ranked variants were also most likely to be replicated across GWAS. For Crohn’s disease the top prioritized variant by ExPecto was rs1174815 (Zhou et al., 2018), yet neither the variant or gene (*IRGM*) has been highly prioritized by any other study focusing on Crohn’s disease. In comparison with other model rankings for Crohn’s disease loci, there are only a handful of genes that have been highly prioritized in more than one study. An example of this is *GSDMB*, a gasdermin gene known to affect apoptosis in epithelial cells. GPRM prioritized this gene, alongside ExPecto prioritizing a variant in *GSDMB* (rs58989791) (Zhou et al., 2018; Gettler et al., 2019). This prioritization has aligned with experimental work recently focusing on *GSDMB* in IBDs, finding an increase in the gene’s expression may have a developmental role for IBDs (Rana and Pizarro, 2019). Another disease that has been prioritized by multiple studies is Alzheimer’s disease, for which models consistently prioritize *APOE* (Mordelet and Vert, 2011; Wang et al., 2013; Deo et al., 2014). However, this questions model training in these studies, as *APOE* has been reported as affecting Alzheimer’s disease as early as 1993 (Schmechel et al., 1993).

An issue with prioritizing variants and genes is the ability to ascertain if the model predictions are accurate. Schubach et al. (2017) address this by prioritizing regulatory variants for both mendelian diseases and complex diseases, for which the mendelian disease variants had been validated with a biomedical literature review. They found hyperSMURF consistently out-performed other methods (Eigen, GWAVA, CADD, and DeepSea) on both mendelian and GWAS data, suggesting minimized risk of overfitting and the potential for ML to be able to generalize across datasets. In terms of performance metrics, Schubach et al. (2017) also explore multiple measurements – F1 score, AUROC, precision, recall, and the area under the precision-recall curve (AUPRC) – however, other studies primarily use AUROC. Whilst AUROC is an excellent metric in many cases, it can be highly misleading for imbalanced datasets like those commonly found in GWAS prioritization (Jeni et al., 2013; Saito and Rehmsmeier, 2015). Precision-recall curves are a popular alternative in cases of extreme class imbalance, with Schubach et al. (2017) applying these in combination with other metrics in a particularly rigorous approach. Studies focused on addressing imbalanced data are important for developing reliable GWAS applications, and continuing to focus on imbalance-aware approaches will reinforce the reliability of model predictions as much as possible *in silico*.

In order to establish model capability past performance metrics, a prioritized variant or gene’s causality can be evidenced with functional follow-up. For example, Lin et al. (2019) developed RegSNPs-Intron which was a random forest prioritizing intronic variants associated to cellular sensitivity to clofarabine-induced cytotoxicity – with the model primarily relying on splicing data. After prioritization they performed ASSET-seq (ASsay for Splicing using ExonTrap and sequencing), which measures the impact of splicing on an intronic variant. They found 63 out of 82 experimentally tested variants had a significant splicing impact in multiple cell lines (Lin et al., 2019), suggesting further directions for functional study and validating the RegSNPs-Intron’s prioritization. Zhou et al. (2018) also performed experimental follow-up, looking at their top prioritized variants with a luciferase assay. This confirmed prioritized variants affect regulatory activity – e.g., variant rs381218 prioritized to affect chronic hepatitis B virus had a significant change in reporter activity, predicted also by ExPecto to impact *HLA-DOA* (Zhou et al., 2018). These functional results improve the interpretation of potential regulatory roles for prioritized loci by validating prioritizations *in vitro*, enabling hypotheses produced by ML to be confirmed and further expanded upon.

## Past and Present Cardiovascular Machine Learning Prioritization

ML approaches for post-GWAS prioritization have been applied over the last decade, with applications providing the projected outputs expected from GWAS with biological insights and translational results. In 2014, Deo et al. (2014) applied OPEN to prioritize 38 phenotypes, many of which were CVD traits. CVD is a particularly appropriate example to investigate, due to its high powered GWAS with thousands of associated loci, presenting a large benefit to gain from ML prioritization. To the best of our knowledge, this is the only ML study that includes CVD traits. OPEN was applied to prioritize BP associated loci, for which several of its highly ranked genes have since been studied in laboratory experiments and leading to insights on biological mechanisms with possible translational impacts. *NPR3* was the second prioritized gene to affect BP by Deo et al. (2014). At the time of prioritization GWAS was one line of evidence showing a relationship between *NPR3* and BP, however, Ren et al. (2018) focused on this gene’s functional roles in vascular smooth muscle. They found variants at this locus were associated with reduced *NPR3* mRNA and changes to chromatin structure, supporting a regulatory role leading to increases in vascular smooth muscle proliferation and suggesting a mechanism which can be a therapeutic target for BP. Overall with examining the top ten prioritized BP genes by Deo et al. (2014) (*ANTXR2*, *NPR3*, *MECOM*, *PLCE1*, *ENPEP*, *PDGFRA*, *CACNB2*, *ARID5B, MRVI1*, and *GUCY1B3*) eight of the associations have been validated by GWAS and mechanisms characterized by experimental work and indicate effects on BP (Rippe et al., 2017; Takeuchi et al., 2018; Giri et al., 2019; Kichaev et al., 2019) – only *ANTXR2*^2^ and *PDGFRA*^3^ have not been validated in recent BP GWAS. The gene *GUCY1B3*, ranked tenth by Deo et al. (2014), and *JAG1* (ranked 11th) have consistently been studied in relation to BP and nitric oxide regulation (Rippe et al., 2019). Rippe et al. (2017) identified both genes as affecting Notch pathway signaling in the aorta of mice, rats and humans – this study provided further insight into each gene’s activity across species. Interestingly, variants at *MRVI1* (ranked eighth) have been found to be genome-wide significant in an arterial stiffness GWAS (Fung et al., 2019), implying a possible relation to BP and opportunity for follow-up investigation such as with colocalization analyses (Kanduri et al., 2019).

OPEN also ranked genes without high prioritization but have since been demonstrated to be important to BP regulation and have clinical significance (Deo et al., 2014). An example of this is uromodulin (*UMOD*), which Deo et al. (2014) prioritized approximately in the middle of their rankings of hundreds of associated genes affecting BP. *UMOD* has been replicated in GWAS (Evangelou et al., 2018) and is a target currently being tested in a clinical trial for its interaction with NKCC2 in hypertension – as *UMOD* genotypes of increased or decreased expression affect salt sensitivity in the kidney and a person’s propensity for hypertension.

Aside from BP, Deo et al. (2014) also report success for other cardiac conditions that have additional evidence and support today. *FLNC* was prioritized as affecting left ventricular diameter. Deo et al. (2014) investigated *FLNC* further in a zebrafish model, finding knocked down *FLNC* showed cardiac abnormalities and hypertrophy, and also found one dilated cardiomyopathy patient (who had no known dilated cardiomyopathy gene mutations) with a splice-site mutation in *FLNC*. This work aligned with *FLNC* gaining functional cardiovascular research attention, with its role in cardiomyopathies also being first discovered in 2014 (Valdes-Mas et al., 2014). This result validates OPEN’s high performance for cardiomyopathies (AUCROCs of 0.88 and 0.96), with its performance ranging from 0.75 to 0.9 for all other cardiac traits. Notably, Deo et al. (2014) used known causal genes as their training examples for cardiomyopathies, unlike the use of GWAS associated genes in the training data for other phenotypes, implying the benefit of using well-curated input data.

The insights into the functions of prioritized genes since 2014 indicate the potential of ML for guiding hypothesis generation, but also outline examples of the experimental work ahead for validating the biological mechanisms of such ranked genes in order to confidently identify drug targets post-GWAS. With 451 BP associated genes gathered by Deo et al. (2014) in comparison to 2,993 validated associated genes in 2019 (Evangelou et al., 2018; Giri et al., 2019), this suggests that re-running OPEN now with updated data would provide interesting results detailing which genes have withstood the test of time in terms of maintaining their ranking.

## Discussion

Machine learning is advancing rapidly but its applications in GWAS are still in their infancy with respect to becoming gold standard methods producing consistently validated biological insights. This review has focused on post-GWAS ML prioritization methodologies ranging from model selection and input features, to performance assessment and output prioritization results. For model selection several studies explore only one algorithm without comparison. Studies using benchmarking comparisons with several models offer a form of standardization for selection, contributing to research transparency which is crucial for work justifying investment in functional study. Recent studies are more frequently incorporating benchmarking comparison showing the development of robust methodology in this field (Isakov et al., 2017; Kafaie et al., 2019; Vitsios and Petrovski, 2019).

The feature curation also needs improved interpretation of selected features and their importance, as current work highlights the need to account for bias within biological features, and the requirement for continued upkeep of biological data. This interlinks with a broader demand for standardized use of recently discovered datatypes, as prioritization studies differ in their resources, hindering the interpretation of model performance. For example with growing epigenomics resources, Cazaly et al. (2019) note this is leading to research using varying standardization methods. How that data is collected and recorded then also affects the reliability of ML methods and comparison of model performances. This point can also be made for models such as ExPecto or iMEGES firstly applying variant prediction which feeds into gene prioritization as a feature (Khan et al., 2018; Zhou et al., 2018), as there is a risk of the predicted features overfitting, and those features then not being reproducible.

There are also datatypes, such as clinical datasets and wider ranges of omics data which are underrepresented in ML prioritization studies. Studies focus on genomic features, however, the contributions of transcriptomic, epigenomic and proteomic data are less frequently investigated. This lack is contrasted by studies solely integrating wide-ranging omics data to calculate GWAS prioritization scores (Ayalew et al., 2012; Ciesielski et al., 2014) – and identifies potential for collaboration with ML to improve data integration methods. To date ML studies highlight the benefits of multi-omic integration, but few directly investigate that need (Merelli et al., 2013; Dai et al., 2019). Building this multi-omic range of data could improve accuracy and provide information specifying not only the most likely causal genes, but the biological functions contributing to their causality. With current data and research there is a disconnect between prioritization of genes and identification of the mechanism that links a feature to gene/variant causality, which could benefit hypothesis specification in functional work.

As high quality disease-specific data becomes increasingly available to fine-tune model training, ML models may become more efficient in the prioritization of heterogenous data to identify the most likely causal disease genes. However, reliance on specific annotations presents a challenge for the prioritization of novel genes and hence novel mechanisms without prior knowledge. More generally, models including data mining features are also susceptible to this issue, as they contribute to a bias for prioritizing already characterized genes in known disease pathways. These already researched genes may be highly ranked not due to impactful biological knowledge but simply due to having a wealth of study. Overall how feature curation is implemented is a key factor to the developing success of ML applications for GWAS, especially when considering imbalanced data where positively labeled disease genes and variants are limited. This highlights the need for high quality gene annotation and disease resources – if features are not accurately researched and curated, the potential for models to accurately prioritize GWAS results will be diminished, ultimately ML methods are limited by the quality and quantity of input training data.

When comparing output prioritizations there is a need to appraise the quality of the training data, understanding which genes/variants are included and how they might impact prediction. For example with the prioritization of *APOE* by models for Alzheimer’s disease (Mordelet and Vert, 2011; Wang et al., 2013; Deo et al., 2014), it could also be argued that this validates the model performance, as this gene is expected to be prioritized. However, the studies prioritizing Alzheimer’s disease genes do not provide their training and testing data to explore this further (Mordelet and Vert, 2011; Wang et al., 2013; Deo et al., 2014), showing the need to improve reproducibility. More recent studies prioritizing different phenotypes are beginning to provide both their data and source code, such as Khan et al. (2018), enabling the development of more accessible and reliable tools. This development is essential for applications to be used and interpreted by non-computer scientists and for the output biological findings to have a traceable reasoning as to why they were prioritized.

On investigating OPEN’s prioritizations and comparing them with more recent research, it emphasizes the potential for post-GWAS ML to give GWAS results a wider-impact contribution to complex diseases. The accuracy of the model across multiple diseases identifies the possibility that one model can be applied to several diseases successfully. Furthermore, the early prioritization of diagnostic genes such as *FLNC* shows the power of ML which, when combined with functional follow-up building biological insights, can lead into translational impacts. However, OPEN also showed genes which upon recent review were mis-prioritized (*UMOD* and *ANTXR2*). This ranking may be due to Deo et al. (2014) using GWAS results as part of their training data with them also noting that their features may be too weak to prioritize genes a part of novel mechanisms for a pathology (Deo et al., 2014). These misjudged genes highlight flaws applicable for all ML models, with reliance on current biological data, requiring that data to be high quality for reliable loci prioritization.

For future applications ML can learn from work such as Deo et al. (2014) in combination with more recent work on larger datasets, e.g., Zhou et al. (2018). Research can develop models aiming to be applied across diseases, and re-used by other researchers, with consideration for the size of present GWAS data, varying datatypes, and feature importance. Doing so could then lead to more accessible, reusable models – for example with source code or web-interfaces that are useable by a wider range of GWAS researchers – and create more globally implemented ML applications for GWAS prioritization, thus accelerating researchers towards the post-GWAS endgame of understanding disease.

With the creation of accessible models, a role for ML prioritization in personalizing medicine can develop. For example, ML could potentially be used to augment genetic risk scores, identifying which genes contribute to a person’s high risk score, and offering more information at the disposal of clinicians. To build ML tools to a clinically acceptable standard, however, requires comparison with other prioritization methods and ensuring model interpretability. One of the most common other methods used in post-GWAS prioritization is network analysis. This method builds networks ranging from the gene to protein level, enabling a flow of information from GWAS to protein and metabolic pathways (Leal et al., 2019). However, studies note that gene networks can contain noise, and the analysis is confounded by its aggregation of GWAS data to the gene level, causing a loss of variant information (Wu et al., 2018; Leal et al., 2019). Machine learning offers an improvement for this with data integration, that can preserve variant information, and with the ability to handle noisy data. Another method identifying causality is Mendelian randomization, although in some cases this can provide a clear illustration of risk, such as the link between homocysteine concentration and stroke risk (Casas et al., 2005), it is limited to high risk variants and independent variables (Haycock et al., 2016). In comparison, unlike other computational methods, the choices ML models make for prioritization are not always clearly available to be understood by the user. However, ML has also been applied in combinational approaches with network modeling (Kafaie et al., 2019) and Mendelian randomization for causal inference (Hemani et al., 2017) to overcome the disadvantages of a singular method. Hybrid approaches such as these highlight the many avenues of ML research to be explored for developing optimal GWAS prioritization. Aside from method comparison, improving data curation, and model benchmarking, the interpretability of models is a critical challenge for future research, and one of the largest obstacles for GWAS prioritization by ML to gain widespread reliable use. Developing model interpretability will involve a strong understanding of not only a model’s mechanics but of feature importance and known disease causing genes given in model training – requiring an interdisciplinary effort to explore the potential of ML post-GWAS prioritization in full.

## Key Concepts

**Supervised learning:** Models learn from labeled training data. Labeled positive and negative examples in training allow a model to practice decision-making before being assessed on new “testing” data.

**Unsupervised learning:** Models learn from unlabeled data. The models recognize patterns between samples that can identify clusters or outliers.

**Semi-supervised learning:** Models use both labeled and unlabeled data during training to perform pattern recognition. This is usually with a larger amount of unlabeled data than labeled data and enables techniques such as positive unlabeled learning.

**Overfitting:** When a model performs well on training data but poorly on test data. Some amount of overfitting is inevitable, but extreme cases can render a model useless.

**Cross-validation:** A procedure for assessing generalization error. Data are split into *k* subsets (or folds) of roughly equal size. Train *k* separate models with each fold held out once for testing. Average error across the *k* trials is reported.

**Class imbalance:** When the ratio of positive to negative labels is far from one, creating less opportunity for a model to learn from the minority class. Imbalance-aware methods perform undersampling or oversampling of majority and minority classes, respectively, to balance the dataset.

**Sensitivity:** The number of true positive samples correctly classified by a model. Also known as the true positive rate or recall.

**Specificity:** The number of true negative samples correctly classified by a model. Also known as the true negative rate or selectivity.

**Precision:** The ratio of true positives to declared positives. Also known as the positive predictive value, and equal to the complement of the false discovery rate.

**AUROC:** Area under the receiver operating characteristic curve, which illustrates the tradeoff between sensitivity and specificity. Can be interpreted as the probability that a classifier will rank a randomly chosen positive instance higher than a randomly chosen negative one.

**AUPRC:** Area under the precision-recall curve, which illustrates the tradeoff between precision and recall; useful when classes are imbalanced.

## Conclusion

Machine Learning is gradually proving itself to be a valuable tool for post-GWAS analysis, as methodology and high quality training data iterates, ML is showing increasingly optimized performance for prioritizing loci. It has begun to output results which have been validated by showing clinical impact. For complex diseases such as CVD, its ability to generate hypotheses has streamlined functional work that has led to biological insights – enabling the unraveling of how the predominantly non-coding associated loci may affect cardiovascular health. However, before ML models can consolidate their role in the post-GWAS analyses, research needs to address several aspects ranging from performance (including model benchmarking and fine-tuning), reproducibility, and accessibility. There also needs to be greater comparison between ML and other prioritization methods in order to understand ML’s place in the post-GWAS pipeline and enable GWAS to truly provide the projected biological insights and translational capability that it has so long promised.

## Author Contributions

HN, PM, MB, and CC outlined and drafted the manuscript. All authors contributed and provided critical review of the manuscript.

## Conflict of Interest

The authors declare that the research was conducted in the absence of any commercial or financial relationships that could be construed as a potential conflict of interest.

## Footnotes

1 https://www.ebi.ac.uk/gwas/

2 https://www.ebi.ac.uk/gwas/genes/ANTXR2

3 https://www.ebi.ac.uk/gwas/genes/PDGFRA
